# Microfluidic-Based Electrical Operation and Measurement Methods in Single-Cell Analysis

**DOI:** 10.3390/s24196359

**Published:** 2024-09-30

**Authors:** Xing Liu, Xiaolin Zheng

**Affiliations:** Key Laboratory of Biorheological Science and Technology, Ministry of Education, Bioengineering College, Chongqing University, Chongqing 400044, China

**Keywords:** microfluidic chips, dielectrophoresis, electroporation, impedance measurement, electrochemical analysis

## Abstract

Cellular heterogeneity plays a significant role in understanding biological processes, such as cell cycle and disease progression. Microfluidics has emerged as a versatile tool for manipulating single cells and analyzing their heterogeneity with the merits of precise fluid control, small sample consumption, easy integration, and high throughput. Specifically, integrating microfluidics with electrical techniques provides a rapid, label-free, and non-invasive way to investigate cellular heterogeneity at the single-cell level. Here, we review the recent development of microfluidic-based electrical strategies for single-cell manipulation and analysis, including dielectrophoresis- and electroporation-based single-cell manipulation, impedance- and AC electrokinetic-based methods, and electrochemical-based single-cell detection methods. Finally, the challenges and future perspectives of the microfluidic-based electrical techniques for single-cell analysis are proposed.

## 1. Introduction

Cellular analysis is crucial for understanding the fundamental processes of biological systems. Minor fluctuations in the local environment or genetic variations can lead to significant cell-to-cell differences within a population, such as in morphology, proliferation, and metabolic rate, demonstrating cellular heterogeneity [1,2]. A detailed study of cellular heterogeneity offers insights into cell cycle progression and tissue origin, which are vital for disease diagnosis, prognosis, therapeutics, and regenerative medicine [3]. The biophysical properties and metabolites or secretions of cells are closely linked to their physiological and pathological states and are commonly used as indicators in single-cell heterogeneity studies [4,5]. Various techniques have been developed to analyze these indicators at the single-cell level. Traditional methods, such as atomic force microscopy [6], optical techniques [7], and patch-clamp methods [8], usually require bulky equipment and have low throughput. Although commercial flow cytometers enable high-throughput analysis, they provide limited cellular information [9]. Additionally, the high cost and reliance on complex optics and fluorescent labeling limit broader applications.

With the advancement of microfabrication techniques, microfluidics has emerged as a versatile tool for single-cell analysis, offering advantages like easy integration, low sample consumption, precise fluid control, and high throughput [4,10,11]. Studies have explored the applications of microfluidic chips for culturing, isolating, and analyzing single cells [12,13]. Microfluidic methods enable the controlled movement and analysis of individual cells in a highly regulated manner. Notably, microfluidic-based electrical techniques are of significant interest, as they provide a label-free, non-invasive method for single-cell analysis, avoiding the need for fluorescent dyes or other invasive mechanical or chemical manipulations. Furthermore, microfluidic-based electrical platforms can be easily miniaturized, eliminating the need for complex instruments. The integration of electrical modules into microfluidic platforms allows for a range of single-cell operations and detections [14], electroporation [15], morphology characterization [16], and metabolite detection [17], all on a single chip.

Given the complexity of single-cell analysis, which often requires preprocessing and pretreatment, there is a growing demand for integrating these functions onto a single chip. In this review, we summarize recent advancements in microfluidic-based electrical methods for single-cell operations and detection, including manipulation, electroporation, and analysis. First, we describe microfluidic-based dielectrophoresis (DEP) techniques for single-cell manipulation, highlighting developments that improve capture efficiency and throughput. Next, we explore microfluidic-based electroporation techniques for extracting cellular components at the single-cell level, focusing on strategies to enhance perforation efficiency. Additionally, we discuss electrical measurements of single cells, including impedance and AC electrokinetic techniques. Finally, we introduce microfluidic-based electrochemical analysis methods and present the challenges and future outlook for microfluidic-based electrical techniques in single-cell analysis.

## 2. Microfluidic-Based Electrical Manipulation of Single Cells

### 2.1. DEP Manipulation of Single Cells

Effective and precise manipulation of single cells is essential to ensure high accuracy in single-cell analysis. Microfluidic-based techniques, including hydrodynamic, electrical, optical, magnetic, and acoustic methods, have been widely adopted for single-cell manipulation due to their minimal sample consumption, precise microenvironment control, and high throughput [14]. Among these, dielectrophoresis (DEP)-based electrical methods have garnered significant attention for their non-invasive, cost-effective, and highly selective capabilities [18].

**Figure 1:**
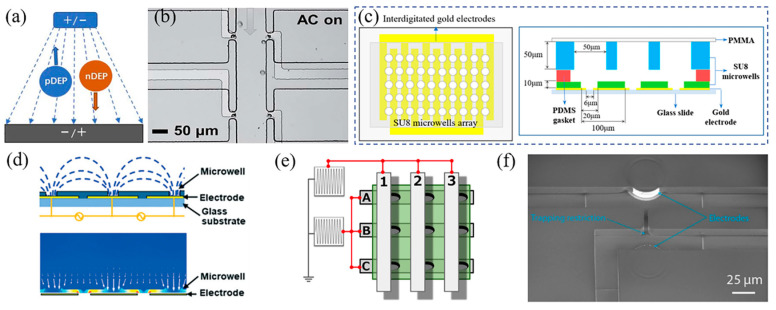
(**a**) Schematic illustration of the nDEP and pDEP. (**b**) Brightfield image of a bipolar electrode-based microfluidic platform for single-cell capture. Reprinted with permission from ref. [28]. Copyright 2019, The Royal Society of Chemistry. (**c**) Schematic illustrations of the interdigitated electrode-based microarray for single-cell capture. Reprinted with permission from ref. [35]. Copyright 2020, The American Chemical Society. (**d**) Schematic illustration and the simulation of the electric field distribution of the DEP-based microarray. Reprinted with permission from ref. [37]. Copyright 2022, The Royal Society of Chemistry. (**e**) Schematic illustration of the three-dimensional DEP-based microarray for selective single-cell capture and release. Reprinted from ref. [50]. Copyright 2021, The Japan Society for Analytical Chemistry. (**f**) Scanning electron microscopy image of the integration of a dynamic trap with the electrodes for single-cell capture. Reprinted from ref. [32].

The microfluidic-based DEP manipulation of single cells relies on cell polarization and the application of a non-uniform electric field. Typically, electrodes are patterned within the microfluidic channel to generate the non-uniform electric field, inducing cell polarization and consequently regulating cell motion or deformation. For a polarizable spherical particle, the DEP force exerted by the non-uniform electric field on the cell can be described as follows [19,20,21]:

(1) $$F_{DEP}=2\pi \varepsilon_{m}r^{3}Re\left[f_{CM}\right]\nabla {\left|E\right|}^{2}$$

where r is the radius of the particle, $\varepsilon_{m}$ is the medium permittivity, $\left|E\right|$ is the root-mean-square electric field, and $Re\left[f_{CM}\right]$ is the real part of the Clausius–Mossoti (CM) factor given by the following:

(2) $$\begin{array}{c} f_{CM}=\frac{\varepsilon_{c}^{*}-\varepsilon_{m}^{*}}{\varepsilon_{c}^{*}+2\varepsilon_{m}^{*}} \end{array}$$

where $\varepsilon_{c}^{*}$ is the complex permittivity of the particle and $\varepsilon_{m}^{*}$ is the complex permittivity of the medium. The magnitude and sign of the $R_{e}\left[f_{CM}\right]$ factor determine the strength and direction of the DEP force applied on the cell, respectively. When $Re\left[f_{CM}\right]>0$, the cell would move toward the higher electric field gradient area, called positive dielectrophoresis (pDEP), while when $Re\left[f_{CM}\right]<0$, the cell would move toward the lower electric field gradient area, called negative dielectrophoresis (nDEP) (Figure 1a). By adjusting the electric field, it is possible to regulate the interplay between DEP forces and other forces exerted on the cell in the microchannel, such as Stokes drag force, enabling a range of single-cell manipulations.

For biological cells in general, it is common to represent them with a single-shell model rather than a simple sphere model, and the effective complex permittivity is given by the following [22]:

(3) $$\tilde{\varepsilon }={\tilde{\varepsilon }}_{mem}\left[\frac{{\left(\frac{R}{R-d_{mem}}\right)}^{3}+2\left(\frac{{\tilde{\varepsilon }}_{cyt}-{\tilde{\varepsilon }}_{mem}}{{\tilde{\varepsilon }}_{cyt}+2{\tilde{\varepsilon }}_{mem}}\right)}{{\left(\frac{R}{R-d_{mem}}\right)}^{3}-\left(\frac{{\tilde{\varepsilon }}_{cyt}-{\tilde{\varepsilon }}_{mem}}{{\tilde{\varepsilon }}_{cyt}+2{\tilde{\varepsilon }}_{mem}}\right)}\right]$$

where d is the thickness of the cell membrane, and the subscripts cyt and mem represent the cell cytoplasm and cell membrane, respectively.

One application of microfluidic-based DEP techniques for single-cell manipulation is the induction of static or cyclic deformation of individual cells by controlling the application of pDEP [23]. Compared to traditional loading techniques, such as conventional atomic force microscopy (AFM), the microfluidic-DEP platform is simpler, more cost-effective, and offers higher throughput. For instance, Qiang et al. used an interdigitated microelectrode array to generate pDEP, guiding individual cells toward the electrode edges and gradually inducing cell deformation by fine-tuning the electric field parameters [24]. Once the electric field is turned off, the cells revert to their original shape. This method provides a straightforward loading-and-unloading approach for assessing the mechanical properties of single cells. By optimizing electrode design and electric field parameters, stable and pronounced cell deformation can be achieved, facilitating precise evaluation of single-cell mechanical properties [25,26].

Another important application of the microfluidic-DEP platform in single-cell manipulation is single-cell trapping. Precise capture and stabilization of single cells are crucial for subsequent analysis to ensure accurate information acquisition [27]. Table 1 comprehensively summarizes microfluidic-based electrical methods for trapping single cells.

The electrode configuration plays a key role in determining the electric field distribution and, consequently, the capture efficiency of single cells [41]. Various electrode designs have been developed to enhance capture efficiency. For example, a four-electrode system can create a more isolated space, generating a nDEP force to focus and stabilize individual cells more effectively than simple planar electrodes [42]. Additionally, corral traps for single-cell capture can be created by fabricating circular non-conductive voids on the surface of a metal coating, utilizing the strong fringing electric field [43]. By modulating the height of the microchannel and the shape of the void, the fringing electric field distribution along the channel’s vertical axis can be optimized, thereby improving trapping efficiency [44,45]. However, limitations such as the short-range electric field gradient and the need for external electrical connections reduce throughput.

A promising approach to enhance the throughput of single-cell capture is the use of bipolar electrode (BPE)-embedded microfluidic systems, which offer a wireless solution by eliminating the need for wire connections [28,46,47]. For example, Li et al. incorporated a BPE array into a microchannel to enable parallel isolation of single cells (Figure 1b) [28]. The BPEs within the microchannel were equipotential, and under AC voltage, maximum electric field intensity was generated at the two ends of the BPEs, while the minimum field was produced in the center. By utilizing pDEP, single cells were captured at the ends of the electrodes, allowing large-scale on-chip single-cell analysis. Similarly, Wu et al. developed a circular BPE array for single-cell capture, where the maximum electric field intensity occurred at the electrode’s edge, and the minimum field at the center [29]. Using nDEP, cells were trapped at the center of the circular electrodes, reducing potential cell damage from high-voltage exposure.

While microelectrodes with various configurations have demonstrated potential for single-cell capture, their reliance on a continuous power supply for cell immobilization and the occurrence of crosstalk between adjacent units limit their broader application in single-cell analysis. To address these challenges, microwell structures on electrodes (MOE) provide an alternative, enabling single-cell capture and immobilization without the need for constant electrical force [30,36,40,48,49]. For example, Bai et al. fabricated an interdigitated electrode array with 3600 microwells positioned between finger electrodes to capture single mouse embryonic fibroblast cells (Figure 1c) [35]. Cells in suspension were captured by pDEP and retained within the microwells long enough for subsequent processes, such as barcoded bead loading for single-cell RNA sequencing. However, due to the weak electric field gradient inside the trap, shallow microwells were required to ensure effective cell capture, which in turn limited the flow rate during cell loading and removal, reducing capture efficiency and risking decreased cell viability due to prolonged capture times.

To improve capture efficiency, Park et al. directly patterned microwell structures onto the bottom electrode, creating a strong localized electric field within the microwell for single-cell capture using pDEP (Figure 1d) [37]. This approach increased the electric field gradient distribution within the channel, enhancing trapping efficiency. The authors noted that trapping efficiency largely depends on microwell size, flow rate, and applied voltage. With optimized parameters, their study achieved over 90% single-cell trapping efficiency and occupancy.

Hata et al. integrated a microwell array into three-dimensional microband electrodes to enable selective single-cell capture and release (Figure 1e) [50]. In their design, two substrates, each containing 12 microband electrodes, were aligned orthogonally and spaced using a double-sided adhesive. Microwells were patterned onto the bottom electrode array, providing cell-sized confinement for long-term single-cell immobilization, which is advantageous for subsequent cell operations and analysis. By adjusting the frequency and voltage applied to each microwell, targeted cells were independently manipulated. Individual cells were trapped using pDEP within one minute and selectively released by switching from pDEP to nDEP via frequency modulation. This platform also allowed single-cell capture at higher flow rates (450 μm/s). Using a similar design, they demonstrated the capture and selective retrieval of antibody-secreting hybridomas by independently controlling the electric field in each microwell for pDEP and nDEP operations [38].

Thiriet et al. combined hydrodynamic microstructures for cell trapping with nDEP for cell release, enabling single-cell trapping and release (Figure 1f) [32]. Due to the flow resistance difference between the main fluid flow and the trap path, cells were captured in the trap. Release was achieved by generating nDEP using electrodes embedded in the microchannel. This design minimized cell damage caused by electrolysis and bubble accumulation on the electrodes. Notably, optimizing the trapping structure to modulate the electric field gradient and hydrodynamic forces could further improve the efficiency of single-cell trapping and release [33].

### 2.2. Microfluidic-Based Electroporation of Single Cells

Single-cell lysis is often a crucial step following cell capture, required to extract cellular contents for subsequent analysis. Compared to commonly used chemical lysis methods, electroporation is a physical technique that disrupts the cell membrane under an electric field, allowing molecules to cross the membrane either irreversibly or reversibly [51,52]. Reversible electroporation, in particular, permits repeated extraction of cellular components without compromising cell viability, enabling real-time downstream analysis. Microfluidic systems, with their confined spaces and precisely controlled microenvironments, facilitate rapid and accurate electroporation of individual cells using low operating voltages [53,54]. Compared to bulk electroporation, which requires high voltage for membrane perforation, microfluidic-based platforms operate at significantly lower voltages, thereby reducing the risk of cell damage, which is favored by reversible single-cell electroporation [15]. Additionally, on-chip electrical methods minimize residual contamination, a common issue with chemical lysis techniques.

Microfluidic-based single-cell electroporation platforms typically use electrode pairs, arranged in either facing or coplanar configurations, to generate a strong electric field for cell perforation. However, high voltage can lead to issues such as electrode degradation, bubble formation, and cell damage [53]. Therefore, optimizing the electric field’s intensity and distribution is crucial for improving both electroporation efficiency and cell viability [15]. To minimize damage, rapid and precise cell targeting is essential. Various strategies, including optimized electrode configurations, microchannel geometries, and fluid dynamics, have been developed to create high-intensity electric fields at low voltages, enhancing single-cell perforation efficiency.

**Figure 2:**
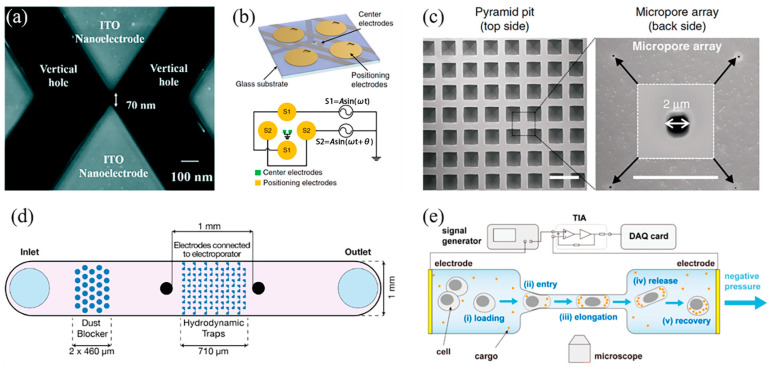
(**a**) Scanning electron microscopy image of triangular coplanar electrodes with nanotips. Reprinted with permission from ref. [55]. Copyright 2020, The Royal Society of Chemistry. (**b**) Schematic illustrations of the sextupole-electrode unit. Reprinted with permission from ref. [56]. Copyright 2020, Zizhong Zhang et al. (**c**) Scanning electron microscopy images of pyramid pit micropore array chip. Reprinted with permission from ref. [57]. Copyright 2020, Zaizai Dong et al. (**d**) Schematic illustration of hydrodynamic microarray-based electroporation platform. Reprinted with permission from ref. [58]. Copyright 2022, Elsevier. (**e**) Schematic illustrations of the constriction channel-based electroporation platform. Reprinted from ref. [59].

Santra et al. developed a coplanar electrode array featuring triangular nanoelectrodes with a 40 nm tip diameter and ~70 nm gap for single-cell electroporation (Figure 2a) [55]. The nanometer-sized gap generates a highly localized electric field, enabling nanoscale electroporation of the cell membrane with an applied voltage of 6 V_PP_, achieving ~98% cell viability. Zhang et al. introduced a sextupole-electrode system for precise single-cell electroporation (Figure 2b) [56]. The electrode unit contains quadrupole electrodes for cell positioning using nDEP force and a pair of microelectrodes with a gap of 7 μm at the center of the quadrupole electrodes for cell perforation and real-time impedance measurement. The real-time monitoring of the cell status allows fast optimization of the electroporation parameters.

Microwell arrays are also used to statically confine individual cells, enabling efficient electroporation [60]. Combining these single-cell trapping structures with micro/nanopores to concentrate the electric field allows precise targeting for single-cell electroporation [57,61,62]. The microarray is positioned between two electrodes, with micro/nano-sized pores at the base of each microwell for electric field concentration. Due to the high resistance of these structures, voltage accumulates around the micro/nanopores, producing a strong electric field at a low operating voltage [53,63]. Additionally, the transmembrane potential across cells in microwells is more uniform compared to bulk electroporation under the same voltage [62]. Dong et al. reported a pyramid pit micropore array chip used to trap individual cells and localize the electric field for single-cell perforation (Figure 2c) [57], using a vacuum system to automate cell patterning. Unlike the manual ‘dipping-trap’ method [61], where cells are trapped by dipping and removing the microarray from suspension, the vacuum-assisted system eliminates manual variability. However, the Joule heating from the ultrafine tip, due to the steep thermal gradient, may cause cell damage and unstable electroporation outcomes [64].

Leveraging microfluidics for precise fluid control and single-cell manipulation, hydrodynamic microtrap arrays [58] and narrow constriction channels [59,65] have been developed for capturing single cells during electroporation. For example, Muralidharan et al. designed a hydrodynamic microarray-based electroporation platform that simultaneously traps and performs localized electroporation on individual cells (Figure 2d) [58]. The microtraps capture cells efficiently by exploiting flow resistance differences between the trap and bulk solution. Additionally, the trap aperture localizes the electric field generated by the electrodes, improving electroporation efficiency and cell viability at a low voltage. Ye et al. developed a constriction microchannel, smaller than the cell diameter, which passively holds cells in place for electroporation (Figure 2e) [59]. A pair of electrodes at either end of the channel perforates cells and evaluates their status via impedance. This device achieved a 96.6% electroporation efficiency for A549 lung cancer cells at 2.5 V_PP_. Ding et al. created a device with a constriction channel and downstream electrodes [59], using the narrow channel to apply a ‘squeezing effect’ that pre-disrupts the cell membrane through high-speed fluid flow. The electric field then perforates the nuclear envelope after the cell exits the constriction. This platform required a lower voltage to achieve the same perforation results as those without constricted channels. The cell deformation and membrane tension induced by fluid flow in the constriction enhance permeability [66].

While microfluidic-based electroporation is highly efficient for single-cell perforation, the precise molecular mechanisms within microfluidic channels remain unclear. The complex intercellular and extracellular environments make selecting electroporation platforms and parameters labor-intensive.

### 2.3. Fabrication of Electrical Microfluidic Devices

As summarized in Table 1, the fabrication of microfluidic electro-manipulation chips typically involves separate processes for the microstructure and microelectrode components. The microstructure is typically fabricated from SU-8 photoresist or polydimethylsiloxane (PDMS), while the microelectrodes are typically fabricated from indium tin oxide (ITO) or gold. The conventional soft lithography technique, which has been reviewed in previous literature [67], can be briefly outlined as the following steps: first, a clean silicon wafer substrate is spin-coated with SU-8 photoresist. The SU-8 is then patterned through photolithography, where it is exposed to UV light and developed, resulting in the formation of SU-8 microstructures. For PDMS microstructures, the SU-8 microstructure serves as a mold. Liquid PDMS is poured over the SU-8 mold, followed by thermal curing. Once cured, the PDMS is peeled off, producing a replica of the microstructure. The fabrication process for typical microelectrodes is RF sputtering or chemical vapor deposition, such as depositing a thin film of ITO or other metal onto a glass substrate and then lithographically patterning it as the required microelectrode shape. In recent years, researchers have been trying to develop new methods of device fabrication [68,69]. In addition to using photolithography and etching techniques, Santra et al. used focused ion beam (FIB) technology [55]. This technique involves the interaction of highly focused, high-energy ions with the material surface, enabling the precise etching of complex triangular nanoelectrode arrays at the nanoscale. Typically, PDMS microstructures are bonded to glass substrates with thin film electrodes after plasma cleaning. For SU-8 microstructures, in some studies, the photoresist can be spin-coated directly on the surface of the ITO electrode and exposed and developed to obtain the desired microwells [37]. SU-8 is difficult to bond to other materials through oxygen plasma cleaning, and therefore double-sided adhesives or mechanical fixtures are commonly used [38].

## 3. Microfluidic-Based Electrical Analysis of Single Cells

The biophysical properties of cells—such as size, morphology, electrical, and mechanical characteristics—are closely linked to their phenotype, functions, and states, making them key indicators for studying cellular heterogeneity [70,71]. Microfluidic-based electrical methods offer a simple, label-free, and non-invasive approach to characterizing the dielectric properties of single cells. These methods allow the extraction of markers like cell diameter, membrane capacitance, conductance, and cytoplasmic conductivity and permittivity, which are useful for identifying the physiological and pathological states of cells [72,73]. Recent microfluidic-based methods for measuring single-cell dielectric properties are summarized in Table 2, primarily categorized into impedance and AC kinetic methods. 

### 3.1. Microfluidic-Based Impedance Measurements of Single Cells

The electrical impedance method is one of the promising techniques for uncovering the biophysical properties of single cells, such as cell size and shape, as well as the mechanical and electrical properties of the cell [73]. The microfluidic-based impedance measurement platform usually contains a microchannel equipped with microelectrodes to generate an AC voltage and record the current response. When a cell passes through the electric field zone, it disturbs the electric field due to the permittivity or the conductivity difference between the cell and the medium [100]. By recording the cell-induced current variation and deciphering the frequency-dependent signal with suitable models, the biophysical properties of the cell could be extracted. The electrical impedance can be calculated as follows:

(4) $$\tilde{Z}=\tilde{V}∕\tilde{I}$$

where $\tilde{Z}$ is the electrical impedance, $\tilde{V}$ is the voltage applied, and $\tilde{I}$ is the current response. The absolute value and phase shift of a complex impedance at different frequencies, along with electrical opacity, are typically calculated to correlate with the biophysical properties of single cells using appropriate equivalent circuit models. Electrical opacity is defined as the ratio of impedance magnitude at high frequencies to that at low frequencies [100].

In the simplest model, a cell consists of a membrane and cytoplasm, with the membrane acting as a capacitor in series with the conductive and capacitive components of the cytoplasm [101]. These two components exhibit distinct dielectric behaviors across different frequency ranges during impedance measurements. At high frequencies, impedance signals reflect intracellular organelles (>10 MHz) and membrane capacitance (2–5 MHz), while at low frequencies (~0.5 MHz), the membrane behaves as an insulator, thereby providing information about cell volume [103].

There are two primary methods for single-cell impedance measurement: electric impedance spectroscopy (EIS) and impedance flow cytometry (IFC). EIS measures the impedance response of immobilized cells across a broad frequency range, allowing detailed cell characterization but with low throughput, while IFC measures impedance in flowing cells, offering high throughput but with lower sensitivity [73]. Microfluidic-based single-cell impedance measurements primarily focus on developing IFC platforms due to EIS’s limitations. However, variations in cell position within microchannels complicate precise impedance measurement in IFC [100]. To address this, different electrode configurations [104,105], microchannel geometries [106,107], and fluid flow-assisted impedance measurement methods [91] have been developed to improve signal-to-noise ratio and measurement reliability.

EIS excels in real-time monitoring and tracking a limited number of cells but is slower than IFC. IFC, while better suited for high-throughput analysis, is typically less sensitive than EIS.

#### 3.1.1. Electrode Configuration

**Figure 3:**
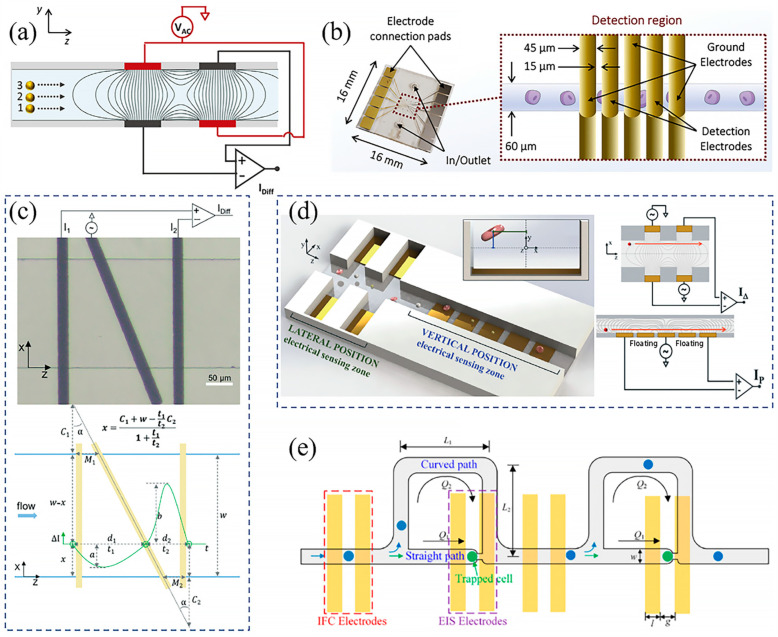
(**a**) Schematic illustration of facing electrode-based impedance measurement system. Reprinted with permission from ref. [104]. Copyright 2020, The Royal Society of Chemistry. (**b**) Schematic illustrations of a five-pair facing electrode system. Reprinted with permission from ref. [74]. Copyright 2019, Elsevier. (**c**) N-shaped coplanar electrode system. Reprinted with permission from ref. [109]. Copyright 2019, The Royal Society of Chemistry. (**d**) Schematic illustration of hydrodynamic microarray-based electroporation platform. Reprinted with permission from ref. [105]. Copyright 2019, The Royal Society of Chemistry. (**e**) Schematic illustration of the combination of EIS and IFC for impedance measurements. Reprinted from ref. [84]. Copyright 2019, The American Chemical Society.

There are two primary microelectrode configurations used for single-cell impedance measurement in microchannels: facing and coplanar. The facing electrode configuration, where electrodes are patterned on opposite inner surfaces of the microchannel, generates a uniform electric field. However, signal deviation due to cell position variations remains a challenge, caused by fringing electric fields. To address this, Caselli et al. designed two pairs of facing electrodes, applying AC voltage diagonally across opposite electrodes while recording the differential current from the other two electrodes (Figure 3a) [104]. The resulting signal, an asymmetric bipolar Gaussian pulse, correlates with the cell’s height in the microchannel. Smaller distances between the cell and recording electrodes result in higher pulse amplitudes, allowing compensation for position-induced signal variations. Spencer et al. developed a device with four pairs of facing electrodes for antimicrobial susceptibility tests [75]. The top two electrodes generated AC voltages at different frequencies, while another set, shifted by 180°, reduced baseline current and improved signal-to-noise ratio. To mitigate crosstalk, Swami et al. incorporated five pairs of facing electrodes, with three used for grounding and two for detection (Figure 3b) [74]. The ground electrodes shield the detection electrodes from neighboring interference, enabling discrimination of pancreatic ductal adenocarcinoma cells based on tumorigenicity. This multi-electrode design allows comprehensive multi-frequency impedance measurements of single cells, offering detailed electrical profiles [108]. However, facing electrode fabrication is complex and prone to alignment issues.

The coplanar electrode configuration, patterned at the bottom of the microchannel, is easier to fabricate and lower in cost compared to the facing electrode setup. However, it produces a non-uniform electric field, making it more sensitive to cell position within the channel, resulting in significant variations of impedance signal. To minimize these position-induced deviations, recent strategies have been developed for coplanar electrode-based platforms. For example, Yang et al. designed an N-shaped coplanar electrode system to measure the lateral position of single cells (Figure 3c) [109]. The system uses a slanted electrode as the excitation source, with two outer electrodes for differential current recording. The varying distances between the electrodes create lateral position-dependent current responses, allowing the determination of cell position. Real et al. expanded on this by developing a system with two detection regions to differentiate both lateral and vertical cell positions (Figure 3d) [105]. In this design, two pairs of electrodes detect lateral position, while a five-electrode system, including floating electrodes, captures vertical position. By analyzing the asymmetric bipolar Gaussian and bipolar double-Gaussian profiles, lateral and vertical positions can be isolated.

To achieve high-resolution impedance spectroscopy in flow cytometry, multiple electrodes have been integrated to apply signals of different frequencies simultaneously. Ai et al. created a position-insensitive seven-electrode system, incorporating two floating electrodes, for high-accuracy leukocyte differentiation (Figure 3e) [80]. This multi-frequency approach enables simultaneous extraction of cell size and electrical properties of the membrane and cytoplasm, improving classification accuracy.

#### 3.1.2. Microchannel Geometry

Another approach to reducing cell position-induced sensing errors is optimizing microchannel geometries. The performance of microfluidic-based impedance detection platforms is highly dependent on channel dimensions [107]. Bilican et al. reported that decreasing the channel height in coplanar electrode-based platforms concentrated the electric field lines, creating a more uniform distribution and minimizing cell position variations [78]. This optimized device successfully discriminated between red blood cells and leukemia cells. The platform’s performance is also influenced by factors such as excitation voltage, solution properties, and cell characteristics. Through optimal design of electrodes, microchannel geometries, and buffer solution, Xie et al. achieved discrimination of submicron-sized bacteria, *B. subtilis* and *E. coli*, and yeast cells [106].

In addition to channel dimensions, specific microstructures like constriction channels and microtraps have been developed to address the position issue in impedance measurements. Constriction microchannels, including straight [110,111,112], crossing [83,90,113], asymmetrical constriction channels [89,113], and multi-constriction channels [114], have been developed to confine the single cells in position for impedance measurements. Using passive force to aspirate the individual cells sequentially into a constriction with a cross-section area smaller than the cell size, the cell can be confined within the constriction, allowing the impedance measurement of single cells. By integrating a straight constriction microchannel between two pairs of electrodes to form an impedance sensing platform, Han et al. characterized the electrical and mechanical properties of individual plant cells simultaneously by analyzing the impedance response and the transit time difference of the deformed cell passing through the constriction channel [82]. The flexible design of constriction structures and reliable circuit models enable the extraction of biophysical properties such as membrane capacitance, cytoplasm conductivity, and viscosity [110]. However, narrow channels pose a risk of clogging and limiting throughput.

Feng et al. combined elements of EIS and IFC by integrating a hydrodynamic cell-trapping microstructure into an IFC platform for efficient single-cell impedance measurements (Figure 3e) [84]. The traps, positioned along a straight path with lower flow resistance than the main curved path, sequentially captured cells. In each detection unit, two pairs of electrodes were placed under the main path and trapping site for IFC and EIS measurements, enabling the acquisition of comprehensive single-cell electrical properties.

#### 3.1.3. Fluid Flow-Assisted Single-Cell Impedance Measurement

To mitigate cell clogging issues in constriction microstructures, fluidic methods such as sheath flow [87,91] and inertial focusing [86,87] have been employed to guide cells along a specific path for impedance measurements. The sheath flow method uses an outer fluid phase to center cells in the channel, while inertial focusing controls cell position by adjusting fluid forces through microstructure design and fluid properties [115,116]. For instance, Petchakup et al. developed a flow-focusing microstructure that modulates viscoelastic sheath flow to deform individual cells from the inner phase [91]. Two pairs of coplanar electrodes placed upstream and downstream of a cross junction measured impedance before and after deformation, allowing the extraction of cell size, membrane, and nucleus properties to assess neutrophils’ biophysical states. Similarly, Tang et al. designed a multifrequency impedance platform with an asymmetric serpentine microchannel and hyaluronic acid as the buffer solution to identify tumor cells [92]. The balance of Dean drag, elastic, and inertial lift forces focused cells into a single train for measurement. However, inertial focusing often requires high flow rates, which may cause cell damage.

### 3.2. Microfluidic-Based AC Electrokinetic Measurement of Single Cells

AC electrokinetic measurements primarily leverage cell polarizability, encompassing methods such as DEP and electrorotation (ROT). DEP induces the movement of polarized cells within a non-uniform electric field, while ROT facilitates cell rotation under electric fields with varying phases [117].

The DEP force, which modulates the interaction between the cell and the electric field, is proportional to the real part of the Clausius–Mossotti (CM) factor, as shown in Equation (1). By adjusting the electric field in relation to the drag force exerted by the fluid on the single cell, cell motion can be effectively controlled. Applying AC voltages with different phase shifts generates a rotational electric field that induces cell rotation. The torque produced by this rotational electric field on a cell can be described as follows: [118]:

(5) TROT=−4πr3εmIm⁡fCME2z

where $\varepsilon_{m}\;$the is the permittivity of the medium, r is the radius of the cell, Im⁡fCM is the imaginary part of the CM factor, $\left|E\right|$ is the root-mean-square magnitude of the applied electric field, and z is a unit vector normal to the electrode surface. The sign of the CM factor determines the direction of the torque. In a steady state, the rotation rate can be expressed as the angular rate $\alpha_{ROT}$ [119]:

(6) αROT = εmξ∕2ηIm⁡fCME2

where η is the dynamic viscosity of the medium, ξ is a scale factor related to the dynamic viscosity and the electric field strength, and the rotation rate is dependent on the imaginary part of the CM factor.

The integration of AC electrokinetic methods with microfluidics enables the measurement of individual cell properties, including mechanical and electrical characteristics [72,120]. This approach allows for the simultaneous extraction of comprehensive electrical properties, such as membrane capacitance, conductance, cytoplasmic conductivity, and permittivity [121]. Typically, quadrupole electrodes serve as the detection unit, where an AC voltage generates non-uniform dielectrophoresis (nDEP) to trap individual cells at the center of the unit [72]. For the single-cell detection, AC voltages with a phase difference of 90° between adjacent electrodes are applied to produce a rotational electric field in order to produce a torque on the cell. By recording the rotation rate of the cell as a function of frequency to form rotation spectra, the electrical properties of the cell can be extracted to be correlated with the cellular lipid content of microalgae cells [42,95] and cellular phenotypes [117,118]. For instance, Trainito et al. used quadrupolar gold electrodes to acquire ROT spectra from mouse ovarian surface epithelial cells at different malignancy stages, revealing an increase in dielectric properties with higher malignancy [117]. 

**Figure 4:**
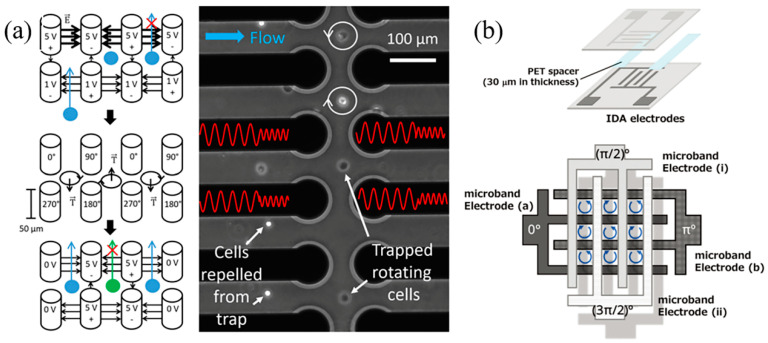
(**a**) Schematic illustration and microscopy image of 3D pillar electrode array for electrorotation measurement. Reprinted with permission from ref. [97]. Copyright 2019, John Wiley and Sons. (**b**) Schematic illustrations of 3D electrode grid array. Reprinted with permission from ref. [98]. Copyright 2020, The Royal Society of Chemistry.

However, the non-uniform electric field from planar electrodes can lead to unstable cell motion during measurements, causing deviations in rotation rate. To address this, Huang et al. developed a single-cell ROT device with four carbon-PDMS side electrodes on planar ITO electrodes, forming a 3D detection unit that stabilizes the cell’s position during measurement [99]. The nDEP forces from the carbon-PDMS and ITO electrodes keep the cell centered, allowing both in-plane and out-of-plane rotation measurements. To enhance throughput, Keim et al. created a 3D pillar electrode array to simultaneously capture and analyze multiple cells via ROT (Figure 4a) [97]. Four pillar electrodes form a sensing unit and an AC signal of 1 V and a 180° phase shift on two input electrodes lowers the DEP barrier, facilitating cell entry into the trap. Meanwhile, a higher DEP barrier is maintained with 5 V on the exit electrodes. Once cells are trapped, the voltage is adjusted to 5 V, and the phase difference is switched to 90° for ROT measurements, achieving a throughput of about 600 cells per hour. The authors noted that further improvements in throughput could be made by enhancing microscope field size and image quality. Kawai et al. developed an electrode grid array with two interdigitated electrode bands to capture and analyze individual cells using nDEP and ROT (Figure 4b) [98]. A weak electric field at the center of the grid retains cells, thereby minimizing friction with the bottom electrode. However, cell aggregation within the grid can reduce single-cell analysis efficiency, which is influenced by cell concentration.

### 3.3. Microfluidic-Based Electrochemical Analysis of Single Cells

**Figure 5:**
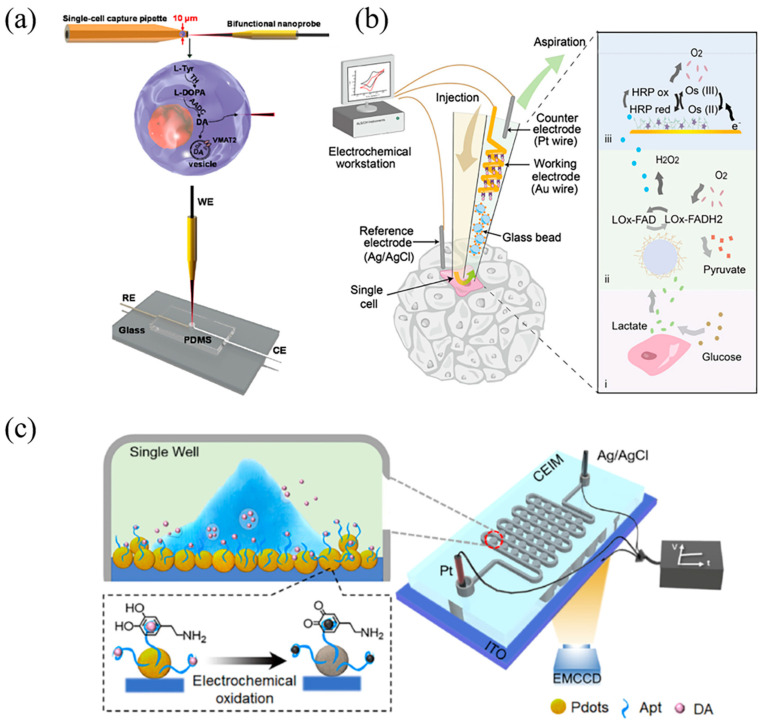
(**a**) Schematic illustration of the bifunctional probe for detection of cytoplasmic dopamine. Reprinted with permission from refs. [126]. Copyright 2021, Elsevier. (**b**) Schematic illustration of the push-pull microfluidic probe for the single-cell detection of lactate. (i–iii) Sample collection and reactions in the detection process. Reprinted with permission from refs. [127]. Copyright 2021, The American Chemical Society. (**c**) Schematic illustration of the hydrodynamic-based ECL microarray for the detection of dopamine at single-cell level. Reprinted with permission from refs. [17]. Copyright 2022, Elsevier.

Variations in the biological and chemical composition of cells significantly influence their functions and contribute to cellular heterogeneity [5,122]. Even cells of the same type can respond differently to external stimuli [1]. Therefore, precise measurement of metabolites or secretions at the single-cell level is essential for investigating this heterogeneity. Electrochemical techniques, known for their high sensitivity, rapid response, and ease of miniaturization, show great promise for single-cell content analysis [123]. Typically, electrochemical sensing involves monitoring analyte-induced changes in electrical signals at detection electrodes, where analytes interact specifically with recognition probes. Given the minute quantities of analytes released by single cells, ultra-sensitive electrochemical methods with high spatial resolution are crucial for cellular analysis. The integration of electrochemical techniques with microfluidics enables controlled detection at the micro/nanoscale, allowing for high spatial resolution and improved signal-to-noise ratios [124,125]. Two primary electrochemical techniques combined with microfluidics for single-cell analysis are micro/nanoelectrode-based methods and electrochemiluminescence (ECL) techniques. Miniaturization of micro/nanoelectrodes reduces background current and minimizes the influence of the electric double layer, enhancing sensitivity for single-cell analysis [124]. For instance, Guo et al. designed a nanometer-sized carbon fiber probe coated with polypyrrole for the electrochemical analysis of intracellular dopamine in a single PC12 cell on a microchip (Figure 5a) [126]. A micropipette captured the cell, allowing the nanoprobe to extract cellular contents without damage, achieving a detection limit of 10 pM for cytoplasmic dopamine. Mao et al. developed a push-pull microfluidic probe with two tapered capillaries to detect lactate released from human cervical cancer cells (CaSki) in real-time (Figure 5b) [127]. By shrinking the detection microchannel into a nanopipette structure with a nanoscale opening and conical shape, signal amplification is enhanced through unique ion current rectification phenomena [128]. Additionally, the nanosized tip allows for intracellular detection of single cells while maintaining cell viability [129]. However, this approach faces challenges such as complex fabrication processes, low throughput, and reliance on manual cell positioning.

Another microfluidic-based electrochemical technique for single-cell analysis is electrochemiluminescence (ECL). ECL involves luminescence triggered by electrochemical reactions, where excited species emit light during electron transfer [130]. This technique offers high sensitivity and low optical background noise without the need for external light sources [131]. By integrating ECL with microstructures, single-cell detection can be achieved with high spatial resolution, as the diffusion of active species is restricted. For example, Xu et al. developed a microwell array to confine individual cells, enabling high-throughput intracellular glucose detection [132]. The microwells limit the lateral diffusion of analytes, and the addition of Triton X-100 breaks the cell membrane, releasing intracellular glucose for ECL detection. This approach was also applied to single-cell cholesterol analysis [133]. Ju et al. designed a hydrodynamic microfluidic chip with immobilized ECL probes in microwells to measure real-time dopamine (DA) release from individual nerve cells [17]. The chip efficiently captures cells in microwells, allowing for high-throughput single-cell analysis. The DA aptamer-conjugated ECL probes enable localized detection of DA with high spatial resolution.

## 4. Conclusions and Perspectives

**Figure 6:**
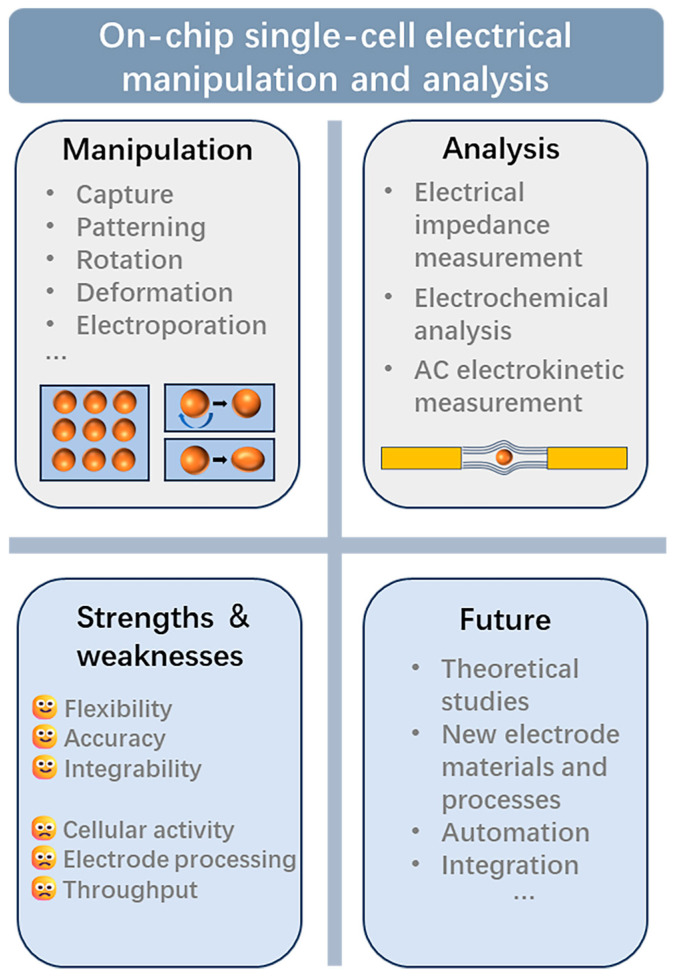
Applications, strengths and weaknesses, and the future of on-chip single-cell electrical manipulation and analysis.

The integration of microfluidics with electrical techniques has shown significant potential for single-cell analysis, providing precise and accurate characterization of cellular heterogeneities. As shown in Figure 6, electrical manipulation and analytical methods have been widely used in the field of single-cell research.

### 4.1. Strengths and Weaknesses

Key strengths include the accuracy of single-cell manipulation, especially controllable patterning, capture, release, and rotation, using a combination of microstructures and DEP, as well as the ability of microfluidic-based impedance cytometry and AC electrokinetic measurement to characterize the dielectric properties of single cells with high throughput. However, several challenges remain. The long-term viability of cells confined in microstructures is compromised by Joule heating and electrolysis induced by high operating voltages. Furthermore, single-cell electroporation still relies on trial-and-error parameter selection, and current impedance flow cytometry techniques are hindered by limited frequency ranges and cell position-induced signal errors, restricting comprehensive intrinsic property analysis. In addition, if the electrical properties of single cells with different biological characteristics are slightly different, the difficulty of precise operation will be greatly increased. However, this is also a common problem for other multi-physics on-chip manipulation and analysis methods.

### 4.2. Opportunities and Threats

There are several opportunities for advancing microfluidic-based single-cell manipulation and analysis. Developing new electrode materials and fabrication methods could improve electrode configurations and microstructures, enhancing the efficiency and scalability of single-cell capture. Exploring the molecular mechanisms underlying electroporation in microfluidic channels may provide reliable criteria for efficient single-cell electroporation, moving beyond the current trial-and-error approach. Integrating multifrequency impedance measurements within microfluidic platforms offers the potential to overcome cell position-induced signal deviations, leading to more accurate and comprehensive characterization of single-cell electrical properties. Further, using machine learning methods for data analysis may be another future direction for extracting the intrinsic electrical properties of single cells [134,135,136]. The development of multiplex analysis arrays for the simultaneous detection of multiple biomarkers at the single-cell level could provide a more holistic evaluation of cellular physiological states. However, there are threats to these advancements. High operating voltages that induce heat and electrolysis remain a threat to cell viability, limiting the effectiveness of long-term analysis. While three-dimensional electrodes can mitigate these effects, their complex fabrication processes limit widespread adoption. Achieving higher throughput for AC electrokinetic and impedance-based single-cell measurements remains a challenge. In conclusion, microfluidics-based electrical platforms are expected to contribute to disease progression and therapeutic research by combining with other methods and integrating multiple manipulation and analysis modules for more efficient and precise on-chip single-cell analysis.

## Figures and Tables

**Table 1:** Summary of microfluidic-based electrical methods for trapping single cells.

Method	Material	Configuration	Cell	Electrical Parameters	Capture Sites	Capture Efficiency/Single-Cell Occupancy	Flow Velocity/Flow Rate	Selectively Release	Ref.
BPE	Electrode:/; structure: PDMS	Electrode: wireless conductor with tip; structure: micropocket	MDA-MB-231; 20 μm	22 V_PP_	40–640	Single-cell occupancy = 84.4%	120 μm/s	NO	[28]
BPE	Electrode: ITO; structure: PDMS	Electrode: wireless conductor of 20 μm diameter circle; structure:/	Yeast cell; 5 μm	500 kHz, 5 V_PP_	1875	Single-cell occupancy = 72%	100 μm/s	NO	[29]
Electroactive microwell array with barriers (EMAB)	Electrode: ITO; structure: SU-8	Electrode: interdigitated array; microwell: 24 μm diameter circle	HeLa cells; -	1 MHz, 5 V_PP_	5000	Capture efficiency = 98%	2 μL/min	NO	[30]
Self-Digitization Dielectrophoretic (SD-DEP) Chip	Electrode: gold; structure: PDMS	Electrode: 50 μm wide parallel array; trap: 15 μm micropocket	Chronic myelogenous leukemia K562 cells; -	1.5 MHz, 5 V_PP_	96	Single-cell occupancy > 90%	/	NO	[31]
Electrode with micropillars	Electrode: Ti/Pt/Ti; structure: SU-8	Electrode: parallel electrode with 15 μm high × 7 μm extrusion; trap: 5 µm aperture	The human T-lymphocytes Jurkat cell line;10 μm	1–20 MHz, 8–10 V_PP_	16	Single-cell occupancy = 90%	/	YES	[32]
Electrode with micropillars	Electrode: gold; structure: PDMS	Electrode: 50 µm wide face-to-face planes; trap: 8 µm × 16 µm with 5 µm gap	Polystyrene microparticles and human leukemia K562 cells; 14–20 μm	1 MHz, 9 V_PP_	36	Single-cell occupancy = 91.34% ± 0.01%	1 μL/min	YES	[33]
Electroactive double-well array (EdWA)	Electrode: ITO; structure: SU-8	Electrode: interdigitated distance, 8 μm; trap-wells (20 μm in diameter) reaction-wells 160	PC3 cells; 15 μm	8 MHz, 5 V_PP_	1464	Capture efficiency = 96% ± 2.8%	2 μL/min	NO	[34]
MOE	Electrode: gold structure: SU-8	Electrode: interdigitated array with 6 μm gap; microwell: 20 μm diameter circle	Human HEK and mouse 3T3 cells; -	10 MHz, 4 V_PP_	3500	Single-cell occupancy = 91.84%	1 μL/min	NO	[35]
MOE	Electrode: ITO; structure: SU-8	Electrode: interdigitated array; microwell: 24 μm diameter circle	Prostate cancer cells (PC3); 22 μm	6 MHz, 10 V_PP_	5554	Capture efficiency = 93.3%	2.5 μL/min	NO	[36]
MOE	Electrode: ITO; structure: SU-8	Electrode:- microwell: 10–30 μm diameter circle	DU-145 cancer cell line; 15.8 μm	0.8–2.6 V_PP_	56,874	Capture efficiency = 97%; single-cell occupancy = 98%	5 μL/min	NO	[37]
MOE	Electrode: ITO; structure: SU-8	Electrode: 40 μm microband; microwell: 16 μm	Hybridomas and mouse myeloma cells	3.0 MHz, 10 V_PP_	144	Single-cell occupancy > 90%	100 μm/s	YES	[38]
MOE	Electrode: ITO, gold; structure: SU-8	A gold-disk electrode stick (electrode diameter of 1.6 mm; square-shaped microwell: 24 μm	Myeloma cells; -	5 MHz, 20 V_PP_	10,000	Cell occupancy = 80–90%	100 μm/s (electrode moving speed)	NO	[39]
MOE	Electrode: gold; structure: SU-8	Electrode: coplanar electrode pairs, 30–120 μm; rectangular microwell: 30/60/90/120 μm × 60 μm	Bakers’ yeast; -	5 MHz, 4 V_PP_	8	Capture efficiency = 100%	20 μL/h	YES	[40]

**Table 2:** Summary of microfluidic-based electrical methods for dielectric property measurement of single cells.

Method	Layout	Frequency	Cell	Measured Parameter	Application	Ref.
Impedance measurement	Five pairs of facing electrodes	0.5, 2, and 50 MHz	Pancreatic ductal adenocarcinoma cells	Electrical opacity	Tumorigenicity identification	[74]
	Four pairs of facing electrodes	5 and 40 MHz	*K. pneumoniae*	Electrical opacity	Antimicrobial susceptibility test	[75]
	Two pairs of facing electrodes	0.5 and 50 MHz	Human neural progenitor cells	Cell diameter and impedance phase	Phenotypic quantification	[76]
	Two pairs of facing electrodes	0.5, 2, 1, and 30 MHz	Raw 264.7 cells	Impedance phase and electrical diameter	Detecting macrophage activation	[77]
	Three coplanar electrodes	0.5 and 10 MHz	U937 cells	Electrical diameter	Cell status detection	[71]
	Three coplanar electrodes	1.5 MHz	Leukemia and human red blood cells	Electrical diameter	Cell discrimination	[78]
	Two pairs of coplanar electrodes	0.5 and 6 MHz	*E. gracilis* cells	Electrical diameter and conductivity of intracellular components	Quantifying cellular component distribution	[79]
	Seven coplanar electrodes	0.26, 1, 8, and 25 MHz	Leukocytes	Electrical opacity, electrical diameter, and impedance phase	Three-part leukocyte classification	[80]
	Five pairs of parallel-facing electrodes	0.5, 5, and 30 MHz	Human red blood cells	Electrical diameter, membrane capacitance, and cytoplasm conductivity	Cell phenotyping	[81]
	Two pairs of coplanar electrodes, straight constriction microchannel	0.5 and 5 MHz	*Arabidopsis* and *Populus* protoplasts	Passing time and electrical opacity	Cell discrimination	[82]
	A pair of coplanar electrodes, crossing constriction channels	100 and 250 kHz	HL-60 cells	Membrane capacitance and cytoplasmic viscosity and conductivity	Cell classification	[83]
	Two pairs of coplanar electrodes, hydrodynamic trap microstructure	1 MHz and 1–1000 kHz	HeLa, HepG2, and A549 cells	Cytoplasm conductivity and membrane capacitance	Cell discrimination	[84]
	Three coplanar electrodes, asymmetric serpentine microchannel	0.5 and 2 MHz	White blood cells, MCF7 cells, and A549 cells	Electrical diameter	Cell phenotyping	[85]
	Three coplanar electrodes, asymmetric serpentine microchannel	0.3 and 1.7 MHz	Leukocytes	Electrical diameter	Cell counting	[86]
	Three coplanar electrodes, hydrodynamic single stream focusing microstructure	0.3 and 1.72 MHz	Lymphocyte cells	Electrical diameter and membrane capacitance	Cell status detection	[87]
	Two pairs of facing electrodes, asymmetric serpentine microchannel	0.5, 2, and 3 MHz	A549 cells and PANC-1 cells	Electrical diameter	Cell counting	[88]
	A pair of planar electrodes, asymmetrical constriction channel	100 and 250 kHz	A549, Hep G2, SW620, AGS, PANC-1, Hela, CAL 27, and HL-60 cells	Membrane capacitance, cytoplasm conductivity, and electrical diameter	Cell classification	[89]
	Two pairs of planar electrodes, crossing the constriction channel	100 and 250 kHz	A549, SACC-83, and SACC-LM cells	Membrane capacitance and cytoplasm conductivity	Cell classification	[90]
	Two pairs of planar electrodes, hydrodynamic cell pinch structure	0.3, 1.72, and 12 MHz	HL-60 cells	Electrical diameter, membrane opacity, and nucleus opacity	Cell counting	[91]
	Three coplanar electrodes, asymmetric serpentine microchannel	0.5, 2, 4, and 6 MHz	White blood cells, A549, MCF7, H226, and H460 cells	Electrical diameter, impedance amplitude and phase shift, and electrical opacity	Cell discrimination	[92]
	Vertical sidewall electrodes	1.0, 1.5, and 2 MHz	Jurkat cells	Impedance magnitude and phase	Cell status detection	[93]
	Aligned parallel microelectrodes in PDMS channel sidewalls	500 kHz and 10 MHz	HeLa cells	Electrical volume and opacity	Cell status detection	[94]
	A pair of coplanar electrodes for every trap	5 MHz	Yeast cells	Impedance magnitude and phase	Cell sorting	[40]
AC electrokinetic measurement	Quadrupolar electrodes	500 kHz (nDEP), 1 MHz (ROT)	Yeast cells	Membrane permittivity and wall conductivity	Detecting the total lipid contents	[95]
	Quadrupolar electrodes	10 kHz (DEP), 0.3–10 MHz (ROT)	Mouse ovarian surface epithelial cell line	Membrane capacitance and cytoplasm conductivity	Detecting cell malignancy	[96]
	Four planar hyperbolic electrodes	1 MHz (nDEP), 37 kHz to 25 MHz (ROT)	Scenedesmus abundans cells	Inner core and wall conductivity and permittivity	Detecting the total lipid contents	[97]
	Eight coplanar electrodes	10 kHz (nDEP), 10 kHz to 100 MHz (ROT)	Sta6 cells	Membrane capacitance and cytoplasm conductivity	Detecting the total lipid contents	[42]
	Three-dimensional sidewall electrodes	100 kHz to 10 MHz	HeLa, A549, HepaRG, MCF7, and MCF10A cells	Membrane capacitance and cytoplasm conductivity	Multiple physical parameter measurements	[98]
	Three-dimensional pillar electrode array	100 kHz (nDEP), 0.01–10 MHz (ROT)	Hela, HEK 293, human T-lymphocyte, and M17 cells	Membrane capacitance and cytoplasm conductivity	Dielectric property measurement	[99]
	3D interdigitated electrode array	100 to 1000 kHz	K562, Jurkat, and THP-1 cells	Membrane capacitance and cytoplasm conductivity	Cell discrimination	[100]
	Four planar electrodes, four 3D sidewall electrodes	0.5 MHz (nDEP), 0.5 MHz (ROT)	Hela cells	-	Three-dimensional cell morphology reconstruction	[101]
	Four microelectrodes arranged on each side of the rectangular microwells	300 kHz	Jurkat cells	Membrane capacitance and conductance	Dielectric property measurement	[102]

## Abbreviations

DEP	Dielectrophoresis
AC	Alternating current
pDEP	Positive dielectrophoresis
nDEP	Negative dielectrophoresis
AFM	Atomic force microscopy
BPE	Bipolar electrode
MOE	Microwell structures on the electrode
PDMS	Polydimethylsiloxane
ITO	Indium tin oxide
FIB	Focused ion beam
EIS	Electric impedance spectroscopy
IFC	Impedance flow cytometry
ROT	Electrorotation
ECL	Electrochemiluminescence technique
DA	Dopamine

## References

[1] Altschuler S.J., Wu L.F. (2010). Cellular heterogeneity: Do differences make a difference?. Cell.

[2] Junker J.P., van Oudenaarden A. (2014). Every cell is special: Genome-wide studies add a new dimension to single-cell biology. Cell.

[3] Pelkmans L. (2012). Cell Biology. Using cell-to-cell variability--a new era in molecular biology. Science.

[4] Chen Y., Zhou Z., Zhu S., Ni Z.H., Xiang N. (2022). Label-free microfluidics for single-cell analysis. Microchem. J..

[5] Evers T.M.J., Hochane M., Tans S.J., Heeren R.M.A., Semrau S., Nemes P., Mashaghi A. (2019). Deciphering Metabolic Heterogeneity by Single-Cell Analysis. Anal. Chem..

[6] Stylianou A., Lekka M., Stylianopoulos T. (2018). AFM assessing of nanomechanical fingerprints for cancer early diagnosis and classification: From single cell to tissue level. Nanoscale.

[7] Galler K., Brautigam K., Grosse C., Popp J., Neugebauer U. (2014). Making a big thing of a small cell—Recent advances in single cell analysis. Analyst.

[8] Aerts J.T., Louis K.R., Crandall S.R., Govindaiah G., Cox C.L., Sweedler J.V. (2014). Patch clamp electrophysiology and capillary electrophoresis-mass spectrometry metabolomics for single cell characterization. Anal. Chem..

[9] Collier J.L. (2000). Flow Cytometry and the Single Cell in Phycology. J. Phycol..

[10] Kulkarni M.B., Ayachit N.H., Aminabhavi T.M. (2023). A Short Review on Miniaturized Biosensors for the Detection of Nucleic Acid Biomarkers. Biosensors.

[11] Sharma V., Mottafegh A., Joo J.U., Kang J.H., Wang L., Kim D.P. (2024). Toward microfluidic continuous-flow and intelligent downstream processing of biopharmaceuticals. Lab Chip.

[12] Wu H., Zhu J., Huang Y., Wu D., Sun J. (2018). Microfluidic-Based Single-Cell Study: Current Status and Future Perspective. Molecules.

[13] Jae-Sung K., Oh J.H. (2018). Microfluidic Technology for Cell Manipulation. Appl. Sci..

[14] Luo T., Fan L., Zhu R., Sun D. (2019). Microfluidic Single-Cell Manipulation and Analysis: Methods and Applications. Micromachines.

[15] Wang F., Lin S., Yu Z., Wang Y., Zhang D., Cao C., Wang Z., Cui D., Chen D. (2022). Recent advances in microfluidic-based electroporation techniques for cell membranes. Lab Chip.

[16] Huang L., Zhao P., Wang W. (2018). 3D cell electrorotation and imaging for measuring multiple cellular biophysical properties. Lab Chip.

[17] Wang N., Ao H., Xiao W., Chen W., Li G., Wu J., Ju H. (2022). Confined electrochemiluminescence imaging microarray for high-throughput biosensing of single cell-released dopamine. Biosens. Bioelectron..

[18] Li M., Anand R.K. (2018). Cellular dielectrophoresis coupled with single-cell analysis. Anal. Bioanal. Chem..

[19] Mohd Maidin N.N., Buyong M.R., Rahim R., Mohamed M.A. (2021). Dielectrophoresis applications in biomedical field and future perspectives in biomedical technology. Electrophoresis.

[20] Kim D., Sonker M., Ros A. (2019). Dielectrophoresis: From Molecular to Micrometer-Scale Analytes. Anal. Chem..

[21] Cha H., Fallahi H., Dai Y., Yuan D., An H., Nguyen N.T., Zhang J. (2022). Multiphysics microfluidics for cell manipulation and separation: A review. Lab Chip.

[22] Valero A., Braschler T., Renaud P. (2010). A unified approach to dielectric single cell analysis: Impedance and dielectrophoretic force spectroscopy. Lab Chip.

[23] Qiang Y., Liu J., Dao M., Suresh S., Du E. (2019). Mechanical fatigue of human red blood cells. Proc. Natl. Acad. Sci. USA.

[24] Qiang Y., Liu J., Dao M., Du E. (2021). In vitro assay for single-cell characterization of impaired deformability in red blood cells under recurrent episodes of hypoxia. Lab Chip.

[25] Hosseini I.I., Moghimi Zand M., Ebadi A.A., Fathipour M. (2021). Cell properties assessment using optimized dielectrophoresis-based cell stretching and lumped mechanical modeling. Sci. Rep..

[26] Hu Q., Wang Z., Shen L., Zhao G. (2022). Label-Free and Noninvasive Single-Cell Characterization for the Viscoelastic Properties of Cryopreserved Human Red Blood Cells Using a Dielectrophoresis-On-a-Chip Approach. Anal. Chem..

[27] Li M., Anand R.K. (2019). Integration of marker-free selection of single cells at a wireless electrode array with parallel fluidic isolation and electrical lysis. Chem. Sci..

[28] Wu Y., Ren Y., Tao Y., Hou L., Jiang H. (2018). High-Throughput Separation, Trapping, and Manipulation of Single Cells and Particles by Combined Dielectrophoresis at a Bipolar Electrode Array. Anal. Chem..

[29] Takeuchi M., Nagasaka K., Yoshida M., Kawata Y., Miyagawa Y., Tago S., Hiraike H., Wada-Hiraike O., Oda K., Osuga Y. (2019). On-chip immunofluorescence analysis of single cervical cells using an electroactive microwell array with barrier for cervical screening. Biomicrofluidics.

[30] Qin Y., Wu L., Schneider T., Yen G.S., Wang J., Xu S., Li M., Paguirigan A.L., Smith J.L., Radich J.P. (2018). A Self-Digitization Dielectrophoretic (SD-DEP) Chip for High-Efficiency Single-Cell Capture, On-Demand Compartmentalization, and Downstream Nucleic Acid Analysis. Angew. Chem. Int. Ed. Engl..

[31] Thiriet P.E., Pezoldt J., Gambardella G., Keim K., Deplancke B., Guiducci C. (2020). Selective Retrieval of Individual Cells from Microfluidic Arrays Combining Dielectrophoretic Force and Directed Hydrodynamic Flow. Micromachines.

[32] Lv D., Zhang X., Xu M., Cao W., Liu X., Deng J., Yang J., Hu N. (2022). Trapping and releasing of single microparticles and cells in a microfluidic chip. Electrophoresis.

[33] Kim S.H., Fujii T. (2016). Efficient analysis of a small number of cancer cells at the single-cell level using an electroactive double-well array. Lab Chip.

[34] Bai Z., Deng Y., Kim D., Chen Z., Xiao Y., Fan R. (2020). An Integrated Dielectrophoresis-Trapping and Nanowell Transfer Approach to Enable Double-Sub-Poisson Single-Cell RNA Sequencing. ACS Nano.

[35] Park J., Komori T., Uda T., Miyajima K., Fujii T., Kim S.H. (2019). Sequential Cell-Processing System by Integrating Hydrodynamic Purification and Dielectrophoretic Trapping for Analyses of Suspended Cancer Cells. Micromachines.

[36] Park J., Park C., Sugitani Y., Fujii T., Kim S.H. (2022). An electroactive microwell array device to realize simultaneous trapping of single cancer cells and clusters. Lab Chip.

[37] Hata M., Suzuki M., Yasukawa T. (2022). Selective retrieval of antibody-secreting hybridomas in cell arrays based on the dielectrophoresis. Biosens. Bioelectron..

[38] Okayama H., Tomita M., Suzuki M., Yasukawa T. (2019). Rapid Formation of Arrayed Cells on an Electrode with Microwells by a Scanning Electrode Based on Positive Dielectrophoresis. Anal. Sci..

[39] Van den Eeckhoudt R., Christiaens A.S., Ceyssens F., Vangalis V., Verstrepen K.J., Boon N., Tavernier F., Kraft M., Taurino I. (2023). Full-electric microfluidic platform to capture, analyze and selectively release single cells. Lab Chip.

[40] Huang K., Cui Z., Lai J., Lu B., Chu H.K. (2022). Optimization of a Single-Particle Micropatterning System With Robotic nDEP-Tweezers. IEEE Trans. Autom. Sci. Eng..

[41] Li Y., Wang Y., Wan K., Wu M., Guo L., Liu X., Wei G. (2021). On the design, functions, and biomedical applications of high-throughput dielectrophoretic micro-/nanoplatforms: A review. Nanoscale.

[42] Li Y., Huang C., Han S.I., Han A. (2022). Measurement of dielectric properties of cells at single-cell resolution using electrorotation. Biomed. Microdevices.

[43] Rahman M.R.U., Kwak T.J., Woehl J.C., Chang W.J. (2021). Quantitative analysis of the three-dimensional trap stiffness of a dielectrophoretic corral trap. Electrophoresis.

[44] Kwak T.J., Lee H., Lee S.W., Woehl J.C., Chang W.J. (2021). Size-Selective Particle Trapping in Dielectrophoretic Corral Traps. J. Phys. Chem. C.

[45] Rahman M.R.U., Kwak T.J., Woehl J.C., Chang W.J. (2021). Effect of geometry on dielectrophoretic trap stiffness in microparticle trapping. Biomed. Microdevices.

[46] Li M., Anand R.K. (2017). High-Throughput Selective Capture of Single Circulating Tumor Cells by Dielectrophoresis at a Wireless Electrode Array. J. Am. Chem. Soc..

[47] Anand R.K., Johnson E.S., Chiu D.T. (2015). Negative dielectrophoretic capture and repulsion of single cells at a bipolar electrode: The impact of faradaic ion enrichment and depletion. J. Am. Chem. Soc..

[48] Menze L., Duarte P.A., Haddon L., Chu M., Chen J. (2022). Selective Single-Cell Sorting Using a Multisectorial Electroactive Nanowell Platform. ACS Nano.

[49] Lei K.F., Ho Y.C., Huang C.H., Huang C.H., Pai P.C. (2021). Characterization of stem cell-like property in cancer cells based on single-cell impedance measurement in a microfluidic platform. Talanta.

[50] Hata M., Suzuki M., Yasukawa T. (2021). Selective Trapping and Retrieval of Single Cells Using Microwell Array Devices Combined with Dielectrophoresis. Anal. Sci..

[51] Stewart M.P., Sharei A., Ding X., Sahay G., Langer R., Jensen K.F. (2016). In vitro and ex vivo strategies for intracellular delivery. Nature.

[52] Tavakoli H., Zhou W., Ma L., Perez S., Ibarra A., Xu F., Zhan S., Li X. (2019). Recent advances in microfluidic platforms for single-cell analysis in cancer biology, diagnosis and therapy. Trends Anal. Chem..

[53] Choi S.E., Khoo H., Hur S.C. (2022). Recent Advances in Microscale Electroporation. Chem. Rev..

[54] Xu X., Wang J., Wu L., Guo J., Song Y., Tian T., Wang W., Zhu Z., Yang C. (2020). Microfluidic Single-Cell Omics Analysis. Small.

[55] Santra T.S., Kar S., Chang H.Y., Tseng F.G. (2020). Nano-localized single-cell nano-electroporation. Lab Chip.

[56] Zhang Z., Zheng T., Zhu R. (2020). Single-cell individualized electroporation with real-time impedance monitoring using a microelectrode array chip. Microsyst. Nanoeng..

[57] Punjiya M., Mocker A., Napier B., Zeeshan A., Gutsche M., Sonkusale S. (2020). CMOS microcavity arrays for single-cell electroporation and lysis. Biosens. Bioelectron..

[58] Chang L., Gallego-Perez D., Chiang C.L., Bertani P., Kuang T., Sheng Y., Chen F., Chen Z., Shi J., Yang H. (2016). Controllable Large-Scale Transfection of Primary Mammalian Cardiomyocytes on a Nanochannel Array Platform. Small.

[59] Dong Z., Jiao Y., Xie B., Hao Y., Wang P., Liu Y., Shi J., Chitrakar C., Black S., Wang Y.C. (2020). On-chip multiplexed single-cell patterning and controllable intracellular delivery. Microsyst. Nanoeng..

[60] Dong Z., Yan S., Liu B., Hao Y., Lin L., Chang T., Sun H., Wang Y., Li H., Wu H. (2021). Single Living Cell Analysis Nanoplatform for High-Throughput Interrogation of Gene Mutation and Cellular Behavior. Nano Lett..

[61] Shokouhi A.R., Aslanoglou S., Nisbet D., Voelcker N.H., Elnathan R. (2020). Vertically configured nanostructure-mediated electroporation: A promising route for intracellular regulations and interrogations. Mater. Horiz..

[62] Yousuff C.M., Tirth V., Zackria Ansar Babu Irshad M., Irshad K., Algahtani A., Islam S. (2021). Numerical Study of Joule Heating Effects on Microfluidics Device Reliability in Electrode Based Devices. Materials.

[63] Muralidharan A., Pesch G.R., Hubbe H., Rems L., Nouri-Goushki M., Boukany P.E. (2022). Microtrap array on a chip for localized electroporation and electro-gene transfection. Bioelectrochemistry.

[64] Ye Y., Luan X., Zhang L., Zhao W., Cheng J., Li M., Zhao Y., Huang C. (2020). Single-Cell Electroporation with Real-Time Impedance Assessment Using a Constriction Microchannel. Micromachines.

[65] Ding X., Stewart M., Sharei A., Weaver J.C., Langer R.S., Jensen K.F. (2017). High-throughput Nuclear Delivery and Rapid Expression of DNA via Mechanical and Electrical Cell-Membrane Disruption. Nat. Biomed. Eng..

[66] Luo Z.Y., Bai B.F. (2019). Solute release from an elastic capsule flowing through a microfluidic channel constriction. Phys. Fluids.

[67] Niculescu A.G., Chircov C., Birca A.C., Grumezescu A.M. (2021). Fabrication and Applications of Microfluidic Devices: A Review. Int. J. Mol. Sci..

[68] Tanwar A., Gandhi H., Kushwaha D., Bhattacharya J. (2022). A review on microelectrode array fabrication techniques and their applications. Mater. Today Chem..

[69] Pattanayak P., Singh S.K., Gulati M., Vishwas S., Kapoor B., Chellappan D.K., Anand K., Gupta G., Jha N.K., Gupta P.K. (2021). Microfluidic chips: Recent advances, critical strategies in design, applications and future perspectives. Microfluid. Nanofluidics.

[70] Phillip J.M., Han K.S., Chen W.C., Wirtz D., Wu P.H. (2021). A robust unsupervised machine-learning method to quantify the morphological heterogeneity of cells and nuclei. Nat. Protoc..

[71] De Ninno A., Reale R., Giovinazzo A., Bertani F.R., Businaro L., Bisegna P., Matteucci C., Caselli F. (2020). High-throughput label-free characterization of viable, necrotic and apoptotic human lymphoma cells in a coplanar-electrode microfluidic impedance chip. Biosens. Bioelectron..

[72] Liang W., Yang X., Wang J., Wang Y., Yang W., Liu L. (2020). Determination of Dielectric Properties of Cells using AC Electrokinetic-based Microfluidic Platform: A Review of Recent Advances. Micromachines.

[73] Zhang Z., Huang X., Liu K., Lan T., Wang Z., Zhu Z. (2021). Recent Advances in Electrical Impedance Sensing Technology for Single-Cell Analysis. Biosensors.

[74] McGrath J.S., Honrado C., Moore J.H., Adair S.J., Varhue W.B., Salahi A., Farmehini V., Goudreau B.J., Nagdas S., Blais E.M. (2020). Electrophysiology-based stratification of pancreatic tumorigenicity by label-free single-cell impedance cytometry. Anal. Chim. Acta.

[75] Spencer D.C., Paton T.F., Mulroney K.T., Inglis T.J.J., Sutton J.M., Morgan H. (2020). A fast impedance-based antimicrobial susceptibility test. Nat. Commun..

[76] Honrado C., Michel N., Moore J.H., Salahi A., Porterfield V., McConnell M.J., Swami N.S. (2021). Label-Free Quantification of Cell Cycle Synchronicity of Human Neural Progenitor Cells Based on Electrophysiology Phenotypes. ACS Sens..

[77] Salahi A., Rane A., Xiao L., Honrado C., Li X., Jin L., Swami N.S. (2022). Single-cell assessment of the modulation of macrophage activation by ex vivo intervertebral discs using impedance cytometry. Biosens. Bioelectron..

[78] Bilican I., Guler M.T., Serhatlioglu M., Kirindi T., Elbuken C. (2020). Focusing-free impedimetric differentiation of red blood cells and leukemia cells: A system optimization. Sens. Actuat B-Chem..

[79] Tang T., Liu X., Yuan Y., Kiya R., Shen Y., Zhang T., Suzuki K., Tanaka Y., Li M., Hosokawa Y. (2022). Dual-frequency impedance assays for intracellular components in microalgal cells. Lab Chip.

[80] Zhong J.W., Tang Q., Liang M.H., Ai Y. (2022). Accurate profiling of blood components in microliter with position-insensitive coplanar electrodes-based cytometry. Sens. Actuat B-Chem..

[81] Salahi A., Honrado C., Rane A., Caselli F., Swami N.S. (2022). Modified Red Blood Cells as Multimodal Standards for Benchmarking Single-Cell Cytometry and Separation Based on Electrical Physiology. Anal. Chem..

[82] Han Z., Chen L., Zhang S., Wang J., Duan X. (2020). Label-Free and Simultaneous Mechanical and Electrical Characterization of Single Plant Cells Using Microfluidic Impedance Flow Cytometry. Anal. Chem..

[83] Liu Y., Wang K., Sun X.H., Chen D.Y., Wang J.B., Chen J. (2020). Development of microfluidic platform capable of characterizing cytoplasmic viscosity, cytoplasmic conductivity and specific membrane capacitance of single cells. Microfluid. Nanofluidics.

[84] Feng Y., Huang L., Zhao P., Liang F., Wang W. (2019). A Microfluidic Device Integrating Impedance Flow Cytometry and Electric Impedance Spectroscopy for High-Efficiency Single-Cell Electrical Property Measurement. Anal. Chem..

[85] Tang D.Z., Chen M., Han Y., Xiang N., Ni Z.H. (2021). Asymmetric serpentine microchannel based impedance cytometer enabling consistent transit and accurate characterization of tumor cells and blood cells. Sens. Actuat B-Chem..

[86] Petchakup C., Tay H.M., Li K.H.H., Hou H.W. (2019). Integrated inertial-impedance cytometry for rapid label-free leukocyte isolation and profiling of neutrophil extracellular traps (NETs). Lab Chip.

[87] Petchakup C., Hutchinson P.E., Tay H.M., Leong S.Y., Li K.H.H., Hou H.W. (2021). Label-free quantitative lymphocyte activation profiling using microfluidic impedance cytometry. Sens. Actuat B-Chem..

[88] Zhu S., Zhang X., Chen M., Tang D., Han Y., Xiang N., Ni Z. (2021). An easy-fabricated and disposable polymer-film microfluidic impedance cytometer for cell sensing. Anal. Chim. Acta.

[89] Zhang Y., Liang H.Y., Tan H.W., Chen D.Y., Wang Y.X., Xu Y., Wang J.B., Chen J. (2020). Development of microfluidic platform to high-throughput quantify single-cell intrinsic bioelectrical markers of tumor cell lines, subtypes and patient tumor cells. Sens. Actuat B-Chem..

[90] Zhang Y., Zhao Y., Chen D., Wang K., Wei Y., Xu Y., Huang C., Wang J., Chen J. (2019). Crossing constriction channel-based microfluidic cytometry capable of electrically phenotyping large populations of single cells. Analyst.

[91] Petchakup C., Yang H., Gong L., He L., Tay H.M., Dalan R., Chung A.J., Li K.H.H., Hou H.W. (2022). Microfluidic Impedance-Deformability Cytometry for Label-Free Single Neutrophil Mechanophenotyping. Small.

[92] Tang D., Jiang L., Xiang N., Ni Z. (2022). Discrimination of tumor cell type based on cytometric detection of dielectric properties. Talanta.

[93] Eades J., Audiffred J.F., Fincher M., Choi J.W., Soper S.A., Monroe W.T. (2023). A Simple Micromilled Microfluidic Impedance Cytometer with Vertical Parallel Electrodes for Cell Viability Analysis. Micromachines.

[94] Yang X., Liang Z., Luo Y., Yuan X., Cai Y., Yu D., Xing X. (2023). Single-cell impedance cytometry of anticancer drug-treated tumor cells exhibiting mitotic arrest state to apoptosis using low-cost silver-PDMS microelectrodes. Lab Chip.

[95] Lin Y.S., Tsang S., Bensalem S., Tsai C.C., Chen S.J., Sun C.L., Lopes F., Le Pioufle B., Wang H.Y. (2021). Electrorotation of single microalgae cells during lipid accumulation for assessing cellular dielectric properties and total lipid contents. Biosens. Bioelectron..

[96] Huang L., Liang F., Feng Y., Zhao P., Wang W. (2020). On-chip integrated optical stretching and electrorotation enabling single-cell biophysical analysis. Microsyst. Nanoeng..

[97] Keim K., Rashed M.Z., Kilchenmann S.C., Delattre A., Goncalves A.F., Ery P., Guiducci C. (2019). On-chip technology for single-cell arraying, electrorotation-based analysis and selective release. Electrophoresis.

[98] Kawai S., Suzuki M., Arimoto S., Korenaga T., Yasukawa T. (2020). Determination of membrane capacitance and cytoplasm conductivity by simultaneous electrorotation. Analyst.

[99] Huang L., Liang F., Feng Y.X. (2019). A microfluidic chip for single-cell 3D rotation enabling self-adaptive spatial localization. J. Appl. Phys..

[100] Zhu S., Zhang X., Zhou Z., Han Y., Xiang N., Ni Z. (2021). Microfluidic impedance cytometry for single-cell sensing: Review on electrode configurations. Talanta.

[101] Daguerre H., Solsona M., Cottet J., Gauthier M., Renaud P., Bolopion A. (2020). Positional dependence of particles and cells in microfluidic electrical impedance flow cytometry: Origin, challenges and opportunities. Lab Chip.

[102] Suzuki M., Kawai S., Shee C.F., Yamada R., Uchida S., Yasukawa T. (2023). Development of a simultaneous electrorotation device with microwells for monitoring the rotation rates of multiple single cells upon chemical stimulation. Lab Chip.

[103] Tang W.L., Tang D.Z., Ni Z.H., Xiang N., Yi H. (2017). A portable single-cell analysis system integrating hydrodynamic trapping with broadband impedance spectroscopy. Sci. China-Technol. Sci..

[104] Caselli F., De Ninno A., Reale R., Businaro L., Bisegna P. (2018). A novel wiring scheme for standard chips enabling high-accuracy impedance cytometry. Sens. Actuat B-Chem..

[105] Reale R., De Ninno A., Businaro L., Bisegna P., Caselli F. (2019). High-throughput electrical position detection of single flowing particles/cells with non-spherical shape. Lab Chip.

[106] Xie X.W., Gong M.L., Zhang Z.W., Dou X.C., Zhou W.B., Li J.S., Zhu M.F., Du Y.H., Xu X.X. (2022). Optimization of an electrical impedance flow cytometry system and analysis of submicron particles and bacteria. Sens. Actuat B-Chem..

[107] Cottet J., Kehren A., van Lintel H., Buret F., Frénéa-Robin M., Renaud P. (2019). How to improve the sensitivity of coplanar electrodes and micro channel design in electrical impedance flow cytometry: A study. Microfluid. Nanofluidics.

[108] Spencer D., Morgan H. (2020). High-Speed Single-Cell Dielectric Spectroscopy. ACS Sens..

[109] Yang D., Ai Y. (2019). Microfluidic impedance cytometry device with N-shaped electrodes for lateral position measurement of single cells/particles. Lab Chip.

[110] Wang K., Liu Y., Chen D.Y., Wang J.B., Chen J. (2022). Development of Microfluidic System Enabling High-Throughput Characterization of Multiple Biophysical Parameters of Single Cells. Ieee Trans. Electron. Devices.

[111] Zhou Y., Yang D., Zhou Y., Khoo B.L., Han J., Ai Y. (2018). Characterizing Deformability and Electrical Impedance of Cancer Cells in a Microfluidic Device. Anal. Chem..

[112] Feng Y., Chai H., He W., Liang F., Cheng Z., Wang W. (2022). Impedance-Enabled Camera-Free Intrinsic Mechanical Cytometry. Small Methods.

[113] Tan H., Wang M., Zhang Y., Huang X., Chen D., Li Y., Wu M.H., Wang K., Wang J., Chen J. (2022). Inherent bioelectrical parameters of hundreds of thousands of single leukocytes based on impedance flow cytometry. Cytom. A.

[114] Yang D., Zhou Y., Zhou Y., Han J., Ai Y. (2019). Biophysical phenotyping of single cells using a differential multiconstriction microfluidic device with self-aligned 3D electrodes. Biosens. Bioelectron..

[115] Yan S., Yuan D. (2021). Continuous microfluidic 3D focusing enabling microflow cytometry for single-cell analysis. Talanta.

[116] Zhou Z., Chen Y., Zhu S., Liu L., Ni Z., Xiang N. (2021). Inertial microfluidics for high-throughput cell analysis and detection: A review. Analyst.

[117] Trainito C.I., Sweeney D.C., Cemazar J., Schmelz E.M., Francais O., Le Pioufle B., Davalos R.V. (2019). Characterization of sequentially-staged cancer cells using electrorotation. PLoS ONE.

[118] Huang L., Fang Q. (2021). Electrical properties characterization of single yeast cells by dielectrophoretic motion and electro-rotation. Biomed. Microdevices.

[119] Tang T., Hosokawa Y., Hayakawa T., Tanaka Y., Li W.H., Li M., Yalikun Y. (2022). Rotation of Biological Cells: Fundamentals and Applications. Engineering.

[120] Henslee E.A. (2020). Review: Dielectrophoresis in cell characterization. Electrophoresis.

[121] Adekanmbi E.O., Srivastava S.K. (2019). Dielectric characterization of bioparticles via electrokinetics: The past, present, and the future. Appl. Phys. Rev..

[122] Walsh A.J., Sharick J.T., Skala M.C. (2019). Imaging intratumoral metabolic heterogeneity. Nat. Biomed. Eng..

[123] Huang Q., Mao S., Khan M., Lin J.M. (2019). Single-cell assay on microfluidic devices. Analyst.

[124] Lu S.M., Peng Y.Y., Ying Y.L., Long Y.T. (2020). Electrochemical Sensing at a Confined Space. Anal. Chem..

[125] Wu Y., Gu Q., Wang Z., Tian Z., Wang Z., Liu W., Han J., Liu S. (2024). Electrochemiluminescence Analysis of Multiple Glycans on Single Living Cell with a Closed Bipolar Electrode Array Chip. Anal. Chem..

[126] Chang Y., Chen Y., Shao Y., Li B., Wu Y., Zhang W., Zhou Y., Yu Z., Lu L., Wang X. (2021). Solid-phase microextraction integrated nanobiosensors for the serial detection of cytoplasmic dopamine in a single living cell. Biosens. Bioelectron..

[127] Zhou L., Kasai N., Nakajima H., Kato S., Mao S., Uchiyama K. (2021). In Situ Single-Cell Stimulation and Real-Time Electrochemical Detection of Lactate Response Using a Microfluidic Probe. Anal. Chem..

[128] Lu S.M., Long Y.T. (2019). Confined Nanopipette-A new microfluidic approach for single cell analysis. Trac-Trends Anal. Chem..

[129] Ruan Y.F., Wang H.Y., Shi X.M., Xu Y.T., Yu X.D., Zhao W.W., Chen H.Y., Xu J.J. (2021). Target-Triggered Assembly in a Nanopipette for Electrochemical Single-Cell Analysis. Anal. Chem..

[130] Oomen P.E., Aref M.A., Kaya I., Phan N.T.N., Ewing A.G. (2019). Chemical Analysis of Single Cells. Anal. Chem..

[131] Zhao W., Chen H.Y., Xu J.J. (2021). Electrogenerated chemiluminescence detection of single entities. Chem. Sci..

[132] Xu J., Huang P., Qin Y., Jiang D., Chen H.Y. (2016). Analysis of Intracellular Glucose at Single Cells Using Electrochemiluminescence Imaging. Anal. Chem..

[133] Xu J., Jiang D., Qin Y., Xia J., Jiang D., Chen H.Y. (2017). C(3)N(4) Nanosheet Modified Microwell Array with Enhanced Electrochemiluminescence for Total Analysis of Cholesterol at Single Cells. Anal. Chem..

[134] Caselli F., Reale R., De Ninno A., Spencer D., Morgan H., Bisegna P. (2022). Deciphering impedance cytometry signals with neural networks. Lab Chip.

[135] Honrado C., McGrath J.S., Reale R., Bisegna P., Swami N.S., Caselli F. (2020). A neural network approach for real-time particle/cell characterization in microfluidic impedance cytometry. Anal. Bioanal. Chem..

[136] Feng Y., Cheng Z., Chai H., He W., Huang L., Wang W. (2022). Neural network-enhanced real-time impedance flow cytometry for single-cell intrinsic characterization. Lab Chip.

